# Genome-Wide Microarray Expression and Genomic Alterations by Array-CGH Analysis in Neuroblastoma Stem-Like Cells

**DOI:** 10.1371/journal.pone.0113105

**Published:** 2014-11-13

**Authors:** Raquel Ordóñez, Gabriel Gallo-Oller, Soledad Martínez-Soto, Sheila Legarra, Noémie Pata-Merci, Justine Guegan, Giselle Danglot, Alain Bernheim, Bárbara Meléndez, Juan A. Rey, Javier S. Castresana

**Affiliations:** 1 Department of Biochemistry and Genetics, University of Navarra School of Sciences, Pamplona, Spain; 2 Institut Gustave Roussy, Villejuif, France; 3 Molecular Pathology Research Unit, Department of Pathology, Virgen de la Salud Hospital, Toledo, Spain; 4 IdiPaz Research Unit, La Paz University Hospital, Madrid, Spain; National Cancer Institute, United States of America

## Abstract

Neuroblastoma has a very diverse clinical behaviour: from spontaneous regression to a very aggressive malignant progression and resistance to chemotherapy. This heterogeneous clinical behaviour might be due to the existence of Cancer Stem Cells (CSC), a subpopulation within the tumor with stem-like cell properties: a significant proliferation capacity, a unique self-renewal capacity, and therefore, a higher ability to form new tumors. We enriched the CSC-like cell population content of two commercial neuroblastoma cell lines by the use of conditioned cell culture media for neurospheres, and compared genomic gains and losses and genome expression by array-CGH and microarray analysis, respectively (in CSC-like versus standard tumor cells culture). Despite the array-CGH did not show significant differences between standard and CSC-like in both analyzed cell lines, the microarray expression analysis highlighted some of the most relevant biological processes and molecular functions that might be responsible for the CSC-like phenotype. Some signalling pathways detected seem to be involved in self-renewal of normal tissues (Wnt, Notch, Hh and TGF-β) and contribute to CSC phenotype. We focused on the aberrant activation of TGF-β and Hh signalling pathways, confirming the inhibition of repressors of TGF-β pathway, as *SMAD6* and *SMAD7* by RT-qPCR. The analysis of the Sonic Hedgehog pathway showed overexpression of *PTCH1*, *GLI1* and *SMO*. We found overexpression of *CD133* and *CD15* in SIMA neurospheres, confirming that this cell line was particularly enriched in stem-like cells. This work shows a cross-talk among different pathways in neuroblastoma and its importance in CSC-like cells.

## Introduction

Neuroblastoma is a neuroendocrine tumor of unknown etiology, derived from primordial neural crest cells which afterwards develop into adrenal medulla and sympathetic ganglia [1]. It is one the most prevalent cancers in childhood and nearly 50% of the cases take place in children younger than two years. The estimated 5-years survival rate is 90% and 50% for patients with non-high-risk and high-risk neuroblastoma, respectively. Most neuroblastomas occur sporadically and develop with very diverse clinical behavior, from spontaneous regression to aggressive malignant progression and resistance to chemotherapy [2], [3].

The actual treatment of neuroblastoma depends on the clinic stage of the tumor, but it is commonly based on radiopharmaceutical therapy in combination with surgery [4]. Current studies and clinical trials are combining conventional chemotherapy with monoclonal antibodies, stem cell transplants and retinoids, but because of the complexity of this pathology, progress remains extremely slow [5].

Some authors propose that this diverse clinical behavior of neuroblastoma might be due to molecular differences in cell subpopulations [6]. The cancer stem cells (CSC) model might be an explanation for this heterogeneous behavior [7]. This model proposes that only a small subpopulation with characteristics of stem cells within the tumor has the ability to proliferate and maintain its growth. Even if a tumoral mass shows a substantial decrease in size in response to therapy, if the CSC are spared, it will regrow leading to a relapse [8], [9]. Some studies propose that CSC operate with the machinery and developmental programs expressed in normal stem cells [10], [11]. There is growing evidence of some signalling pathways involved in self-renewal of both normal and tumor tissues as Wnt [12], [13], Sonic Hedgehog (Hh) [14], [15], Notch [16], [17] and Transforming Growth Factor Beta (TGF-β) [18]–[20] signalling pathways, that might contribute to tumorigenesis when deregulated [14], [21].

In this line, the development of new therapies based in molecular targets may be of great value for the treatment of neuroblastoma. The observation of some pathways acting on multiple levels to promote the development of neuroblastoma and CSC subpopulation, has prompted new therapeutic strategies to treat not only this neoplasm but other brain and nervous system tumors [2], [18], [22].

Therefore, this project performs a genomic analysis of CSC-like by array-CGH and expression array, with special focus on altered signalling pathways that might explain the stem cell phenotype of the CSC subpopulation. The aim of this study is to provide a powerful tool to open up new targets for therapy or redirect current cancer treatments towards CSC in order to achieve total elimination of tumor cell population and improve treatment effectiveness.

## Materials and Methods

### Cell lines culture

Two commercial neuroblastoma-derived cell lines were used: SK-N-DZ cell line (ATCC N°CRL-2149) provided by the American Type Culture Collection (ATCC, Manassas, VA, USA) and SIMA cell line (DSMZ N° ACC 164) provided by the German Collection of Microorganisms and Cell Cultures (DSMZ, Braunschweig, Germany). Both were grown as monolayer with DMEM/GlutaMAX medium supplemented with 10% fetal bovine serum, 5% non-essential aminoacids, 1% penicillin/streptomycin and 0.1% amphotericin B. To obtain the neurospheres cultures in order to enrich them in CSC-like cells, after chemical dissociation of SK-N-DZ and SIMA cell lines, 5×10^5^ cells were transferred to 25 cm^2^ flasks (positioned vertically) and grown in suspension with 5 ml of selective medium: DMEM-F12/GlutaMAX plus 1% penicillin/streptomycin and 0.1% amphotericin B, supplemented with Epidermal Growth Factor (EGF) (20 ng/ml), Fibroblast Growth Factor Basic (FGFb) (20 ng/ml) and B27 Supplement (1X). All cultures were maintained at 37°C in a humidified atmosphere of 5% CO_2_/95% air.

### DNA/RNA isolation and reverse transcription

DNA from 1×10^6^ cells pellet was extracted using Wizard Genomic DNA Purification Kit (Promega, Madison, WI, USA) and total RNA from 2×10^6^ cells pellet, employing Illustra RNAspin Mini Kit (GE Healthcare, Buckinghamshire, UK) following manufacturer's instructions in both cases. DNA and RNA samples were stored at −20°C and −80°C until utilization, respectively. DNA and RNA concentrations were measured with a SmartSpec Spectophotometer (Bio-Rad, Hercules, CA, USA). Reverse transcription was carried out from 2 µg of total RNA (previously denatured at 65°C for 5 min) with a mixture of 0.5 µg of random primers, 0.5 mM dNTPs, 10 mM DTT, First-Strand buffer 1X and 200U SuperScript II Reverse Transcriptase (Invitrogen, Carlsbad, CA, USA) in a total volume of 40 µl. The mixture was incubated for 10 min at 25°C, 50 min at 42°C and 15 min at 70°C, followed by chilling on ice. The cDNA was diluted 1/5 and stored at −80°C until utilization.

### Array-CGH

DNA samples were analyzed using a 244K microarray (Agilent Technologies, Santa Clara, CA, USA). Oligonucleotide aCGH processing was performed as detailed in the manufacturer's protocol (version 4.0; http://www.agilent.com). Data were extracted from scanned images using feature extraction software (version A.8.5.3, Agilent). Raw data text files from the latter were then imported for analysis into CGH Analytics 3.4.40. Aberrations were detected with the ADM2 algorithm and filtering options of a minimum of 5 probes and abs(log2Ratio)>0.3. Aberration segments were individually reviewed using build 35, hg17 of UCSC. Anomalies that were localized to regions with high-copy repetitive or GC-rich DNA sequences including telomeric regions were excluded. Gains and losses for the oligonucleotide dataset were defined as a linear ratio ≥1.2 or ≤0.8, respectively. High and low-level amplification events were defined as a linear ratio ≥4 or 2<ratio<4, respectively. The data are described in accordance with MIAME guidelines and have been deposited in ArrayExpress under E-MTAB-2866 accession number. All the analysis and statistics related with the array-CGH was performed at Institut Gustave Roussy by the bioinformatics team.

### Microarray experiment, data normalization and analysis

SurePrint G3 human Gene Expression Microarray Kit (Agilent, ID:028004) was used to analyze the transcriptional profiles of all samples. This array contains 42,404 60-mer oligonucleotides targeting 27,988 Entrez Gene RNAs and 7,419 lincRNAs (long intergenic non coding RNAs) in an 8×60K format slide (each slide contains 8 arrays of 60,000 features with 1440 spots reserved for internal quality control). The transcripts used can be checked in the EMBL-EBI Database (http://www.ebi.ac.uk/arrayexpress/arrays/A-GEOD-14550).

Array performance and analysis were carried out at the Institut Gustave Roussy, using the Agilent Feature Extraction software. Data sets were normalized using quantile normalization, and fold change between cell line and neurospheres was calculated for each gene. The whole dataset was filtered by intensity of the probes. Spots with too high intensities (saturating intensities) and irregular spots (spots inside whose neighbouring pixel showed very different intensities) were not further considered. Likewise, genes with a signal-to-noise ratio lower than 6 fold the average standard deviation observed for all negative control probes were excluded. Finally, >1 fold-change between normalized gene expression measured in neurospheres (or CSC-like cells) versus standard cell lines was used as the criteria to select differentially expressed genes.

The list of differentially expressed genes in both cell lines was imported and classified by DAVID (Database for Annotation, Visualization and Integrated Discovery, http://david.abcc.ncifcrf.gov/), version 6.7 [23]–[25]. Enriched categories in biological processes and molecular function were defined by a p value <0.05 and more than 2 genes differentially expressed in each category. After the enrichment analysis, a classification by PANTHER (Protein ANalysis THrough Evolutionary Relationships, www.pantherdb.org/) Data Base [26]–[28] was performed. Finally, a deeper pathway analysis was carried out by comparing the same set of genes with the NCBI BioSystems Database (http://www.ncbi.nlm.nih.gov/biosystems). Microarray expression data are available at ArrayExpress with accession number E-MTAB-2867.

### Real-Time polymerase chain reaction (RT-qPCR)

A first screening of our primer library was performed according to the microarray data and pathway analysis. 44 pairs of primers were selected and aligned with the mRNA and DNA sequences obtained from RefSeq (http://www.ncbi.nlm.nih.gov/RefSeq/) and UCSC Genome Browser (http://genome.ucsc.edu/). A deeper analysis of each pair of primers was performed with Primer3 (http://primer3.sourceforge.net/) and Primer Blast (http://www.ncbi.nlm.nih.gov/tools/primer-blast/) in order to obtain product length, %GC, melting temperature (Tm), self-complementarity or possible products on unintended templates. After the computational analysis, 17 out of 44 primers (Table 1) were selected for further studies. RT-qPCRs were performed in order to define the optimal Tm for each pair of primers. The melting curves obtained were analyzed to check amplification efficiency in each temperature, and detect additional peaks displaced from the desired amplicon peak (determined by agarose gels checking size and identifying possible intron amplifications). For the quantitative PCR reaction, 2.5 µl of the generated cDNA was added to a PCR mix containing 12.5 µl SYBR Green Supermix (Bio-Rad, Hercules, CA, USA) and 12.5 pM forward and reverse primers, in a total volume of 25 µl. The reaction was performed according to the following protocol: 10 min at 95°C, followed by 40 cycles of 15 s at 95°C, 30 s at the specific Tm for each primer (Table 1) and 20 s at 72°C. A melting curve was added at the end of the protocol as a quality control. All samples were run in triplicate and three independent experiments were carried out. Gene expression levels between samples were normalized using the expression levels of the *HPRT1* gene. Relative gene expression was analyzed according to the 2^−ΔΔCt^ method [29].

**Table 1:** Primer sequences.

Gene	Primer sequence (5′- 3′)	Ref Seq Acc. #	Tm (°C)	Product (bp)
*PTCH*	F: GGCAGCGGTAGTAGTGGTGTTC	NM_000264.3	64	191
	R: TGTAGCGGGTATTGTCGTGTGTG			
*SHH*	F: AGGCTGATGACTCAGAGGTGT	NM_000193.2	64	144
	R: GCCCTCGTAGTGCAGAGACT			
*GLI1*	F: TTCCTACCAGAGTCCCAAGT	NM_005269.2	64	185
	R: CCCTATGTGAAGCCCTATTT			
*GLI2*	F: GCCATATGTGTGTGAGCACGA	NM_005270.4	64	110
	R: TCTTGCAGATGTAGGGTTTCTCG			
*GLI3*	F: CGAACAGATGTGAGCGAGAA	NM_000168.5	64	185
	R: TTGATCAATGAGGCCCTCTC			
*SMO*	F: CAGCTTCCGGGACTATGTGCTATG	NM_005631.4	64	101
	R: GAAGGCTCGGGCGATTCTTG			
*SUFU*	F: CCTCCAGATCGTTGGTGTCT	NM_016169.3	58	132
	R: CCCCTCCGCATGTCAGTT			
*TGFBR1*	F: CGTCAGGTTCTGGCTCAGGTT	NM_004612.2	58	184
	R: TCTGCCTCACGGAACCACGAA			
*TGFBR2*	F: ACGTTCAGAAGTCGGATGTGG	NM_001024847.2	64	142
	R: TGTGGAAACTTGACTGCACCGT			
*TFGB2*	F: GCCTGAACAACGGATTGAGC	NM_001135599.2	64	124
	R: ATCGAAGGAGAGCCATTCGC			
*TGFB3*	F: ATGATGATTCCCCCACACCG	NM_003239.2	64	153
	R: CTTCCAGCCCAGATCCTGTC			
*BAMBI*	F: AGCTACATCTTCATCTGGCTGC	NM_012342.2	64	187
	R: CATGGGTGAGTGGGGAATTTG			
*SMAD6*	F: AATCTCCGCCACCTCCCTAC	NM_005585.4	64	131
	R: GAATTCACCCGGAGCAGTGA			
*SMAD7*	F: CCAACTGCAGACTGTCCAGATGCT	NM_005904.3	58	136
	R: ATGCCACCACGCACCAGTGT			
*JAG1*	F: ATGGGCCCCGAATGTAACAG	NM_000214.2	64	117
	R: ATCACAGTACAGGCCTTGCC			
*CD133*	F: TCCGGGTTTTGGATACACCCTA	NM_001145847.1	64	155
	R: CTGCAGGTGAAGAGTGCCGTAA			
*CD15*	F: AGGAGGTGATGTGGACAGCG	NM_002033.3	58	160
	R: AACTACGAGCGCTTTGTGCC			
*HPRT*	F: TGACACTGGCAAAACAATGCA	NM_000194.2	64/58	94
	R: GGTCCTTTTCACCAGCAAGCT			

F: foward and R: reverse, reference sequences accession number (RefSeq Acc. #), optimal melting temperature (Tm) in Celsius (°C) and size of the PCR product in base pairs (bp).

### Statistical analysis

The statistical analysis of arrays are detailed in the description for each technique. The data of gene expression measured by RT-qPCR was graph as the mean ± standard deviation. The 2^−ΔΔCt^ values were analysed for normality distribution and homogeneity of variance. Since the data did not show normality distribution, the statistical significance between groups was compared by pairs (the expression level of each gene in standard cultures vs neurospheres cultures) with the non-parametric Mann-Whitney U test. The significance level was set as p<0.05. The statistical program GraphPad Prism for Windows version 5.04 was used.

## Results

### Genomic profile by array-CGH analysis

**Figure 1:**
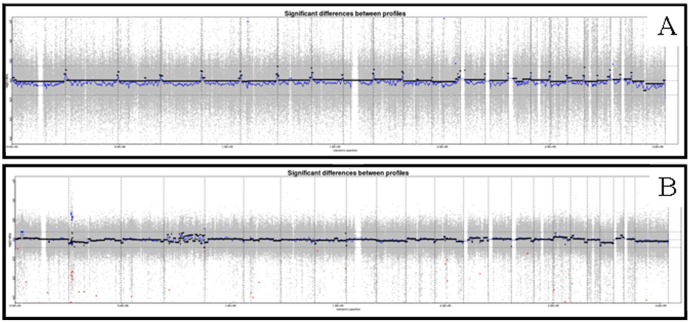
Whole chromosome plots. Array-CGH from SK-N-DZ (A) and SIMA (B) cell lines. The X-axis represents the chromosomes while the Y-axis represent the normalize log2 ratio fluorescence intensity thresholds −1 (loss) and 1 (gain). The results show gains and losses of small chromosomal regions.

To identify the potential common chromosomal alterations indicating possible zones implicated in the CSC-like phenotype, an array-CGH analysis was performed. In the case of SK-N-DZ, we only found six chromosomal regions amplified and no deleted region. SIMA cell line showed more regions of gain and loss, specifically 10 and 30 regions, respectively (data summarized in Table 2). Notwithstanding we did not find any region that was lost or gained in both cell lines and the significance was found only in some points of the genome, we identified numerous chromosomal sequences with copy number variants (CNV) both in SK-N-DZ and SIMA cell lines (Table 2). Whole genome profiles are presented in Figure 1. Among the genes that were included in these regions identified by the array-CGH, the analysis by PANTHER did not show any pattern or group of genes directly related with signalling pathways involved in the CSC-like phenotype. Only a few genes were associated with pathways already described in tumorigenesis and stemness (as p53) but were not principal effectors and its position was downstream, including genes that were related with different regulation points and pathways (data not shown) making the analysis difficult. Despite these results, we noticed that several amplified areas included CpG islands (Table 2). Using the miRBase of the University of Manchester (www.mirbase.org), we were able to identify the microRNAs included in the gained areas of the chromosomes 1, 2 and 12 (Table 3).

**Table 2:** Overview on gained (G) and lost (L) chromosomal regions detected by array-CGH.

Cr:start:end	Size (Kb)	Citogenetic localization	Status	log_2_ ratio	CNV	miRNA	CpGIsl	Genes
**SK-N-DZ**								
1:10532178:10567923	35.75	1p36.22	G	2.50	2	-	2	*DFFA, PEX14*
6:33258397:33259821	1.43	6p21.32	G	1.51	-	-	-	*PFDN6, RGL2*
8:145921822:145943761	21.94	8q24.3	G	2.66	2	-	2	
12:57591347:57628354	37.01	12q13.3	G	1.58	-	-	4	*LRP1, NXPH4, SHMT2*
12:110214797:110344458	129.66	12q24.11	G	0.43	2	1	4	*TRPV4, GLTP, TCHP*
21:10701592:11087870	386.28	21p11.2	G	0.42	98	-	3	*BAGE2, TPTE, BAGE5, BAGE*
**SIMA**								
1:27739420:37252011	9.51×10^3^	1p36.11	G	0.20	206	5	163	*WASF2, AHDC1, FGR, IFI6, FAM76A, STX12, PPP1R8, SCARNA1, C1orf38, RPA2, SMPDL3B, XKR8, EYA3, SPCS2, PTAFR, DNAJC8, ATPIF1, SESN2, MED18, PHACTR4, RCC1, TRNAU1AP, SNORD99, SNORA61, SNORA44, SNORA16A, RAB42, TAF12, RNU11, GMEB1, YTHDF2, OPRD1, EPB41, TMEM20*
2:10490105:10743367	253.26	2p25.1	G	0.68	15	-	5	HPCAL1, ODC1, NOL10
2:11755630:11905792	150.16	2p25.1	G	0.65	6	-	5	GREB1, NTSR2, LPIN1
2:11959615:12260468	300.85	2p25.1	G	0.64	14	1	-	LPIN1
2:14397903:14490030	92.13	2p24.3	G	0.51	5	-	-	
2:14726070:15319138	593.07	2p24.3	G	0.51	13	-	1	FAM84A, NBAS
2:15743981:15893191	149.21	2p24.3	G	0.58	2	-	-	DDX1
2:17683283:17860174	176.89	2p24.2	G	0.60	4	-	2	RAD51AP2, VSNL1, SMC6
2:18422521:18741209	318.69	2p24.2	G	0.56	11	-	-	NT5C1B, RDH14
4:190747562:190817136	69.58	4q35.2	G	0.28	52	-	2	
1:12169416:12228108	58.69	1p36.22	L	−0.23	2	-	1	TNFRSF8, TNFRSF1B
1:150530677:150531841	1.17	1q21.3	L	−1.35	-	-	-	ADAMTSL4
1:51760113:51770840	10.73	1p32.3	L	−1.08	-	-	-	TTC39A
12:48380644:48389191	8.55	12q13.11	L	−0.62	-	-	-	COL2A1
12:57593050:57606248	13.20	12q13.3	L	−0.51	-	-	1	LRP1
12:57925800:57926884	1.08	12q13.3	L	−1.05	1	-	-	DCTN2
13:76141342:76151800	10.46	13q22.2	L	−0.87	1	-	-	UCHL3
15:74635420:74707504	72.08	15q24.1	L	−0.35	2	-	3	CYP11A1, SEMA7A
17:3594077:3595092	1.02	17p13.2	L	−1.28	1	-	-	P2RX5
17:56283529:56284308	0.78	17q22	L	−1.59	-	-	-	MKS1
18:7013911:7023395	9.48	18p11.31	L	−1.12	-	-	-	LAMA1
2:10923316:10924881	1.57	2p25.1	L	−1.60	-	-	-	ATP6V1C2, PDIA6
2:11312145:11718686	406.54	2p25.1	L	−0.91	18	1	3	PQLC3, ROCK2, E2F6, GREB1
2:128393779:128398576	4.80	2q14.3	L	−1.44	5	-	-	MYO7B, LIMS2
2:13527222:13726873	199.65	2p24.3	L	−0.84	22	-	-	-
2:16396225:16608842	212.62	2p24.3	L	−0.94	12	-	-	-
2:16719258:17667981	948.72	2p24.2	L	−1.02	34	-	-	FAM49A
2:17864935:18277059	412.12	2p24.2	L	−0.81	26	-	3	SMC6, GEN1, MSGN1, KCNS3
2:47273218:47294502	21.29	2p21	L	−1.33	-	-	-	TTC7A, CALM2
2:71191955:71205512	13.56	2p13.3	L	−1.33	-	-	1	ATP6V1B1
2:71246290:71263795	17.51	2p13.3	L	−1.83	3	-	-	OR7E91P
3:49155018:49156532	1.51	3p21.31	L	−1.45	-	-	-	USP19
4:190824776:190874516	49.74	4q35.2	L	−0.29	6	-	1	FRG1
4:25780740:25785945	5.21	4p15.2	L	−1.28	-	-	-	SEL1L3
6:33258397:33260455	2.06	6p21.32	L	−1.38	-	-	-	PFDN6, RGL2
6:43258794:43266869	8.08	6p21.1	L	−1.48	1	-	-	SLC22A7
6:71018884:71038982	20.10	6q13	L	−1.09	-	-	-	-
8:11415447:11565691	150.25	8p23.1	L	−0.29	40	-	9	BLK, GATA4
8:143008634:143243125	234.49	8q24.3	L	−0.74	18	-	2	-
X:53224324:53225545	1.22	Xp11.22	L	−1.55	1	-	-	KDM5C

Chromosome:Chromosomal start position:Chromosomal end position (Cr:start:end). Number of polymorphisms or Copy Number Variants (CNV) in the region. Number of miRNA (miRNA) contained in the region. CpG islands (CpGIsl).

**Table 3:** MicroRNA identified in array-CGH analysis.

Citogenetic localization	miRNA
**SK-N-DZ**	
12q24.11	hsa-mir-4497
**SIMA**	
1p36.11	hsa-mir-4420; hsa-mir-4254; hsa-mir-5585; hsa-mir-3605; hsa-mir-552
2p25.1	hsa-mir-4262; hsa-mir-4429

### Gene expression profiles by microarray analysis

**Figure 2:**
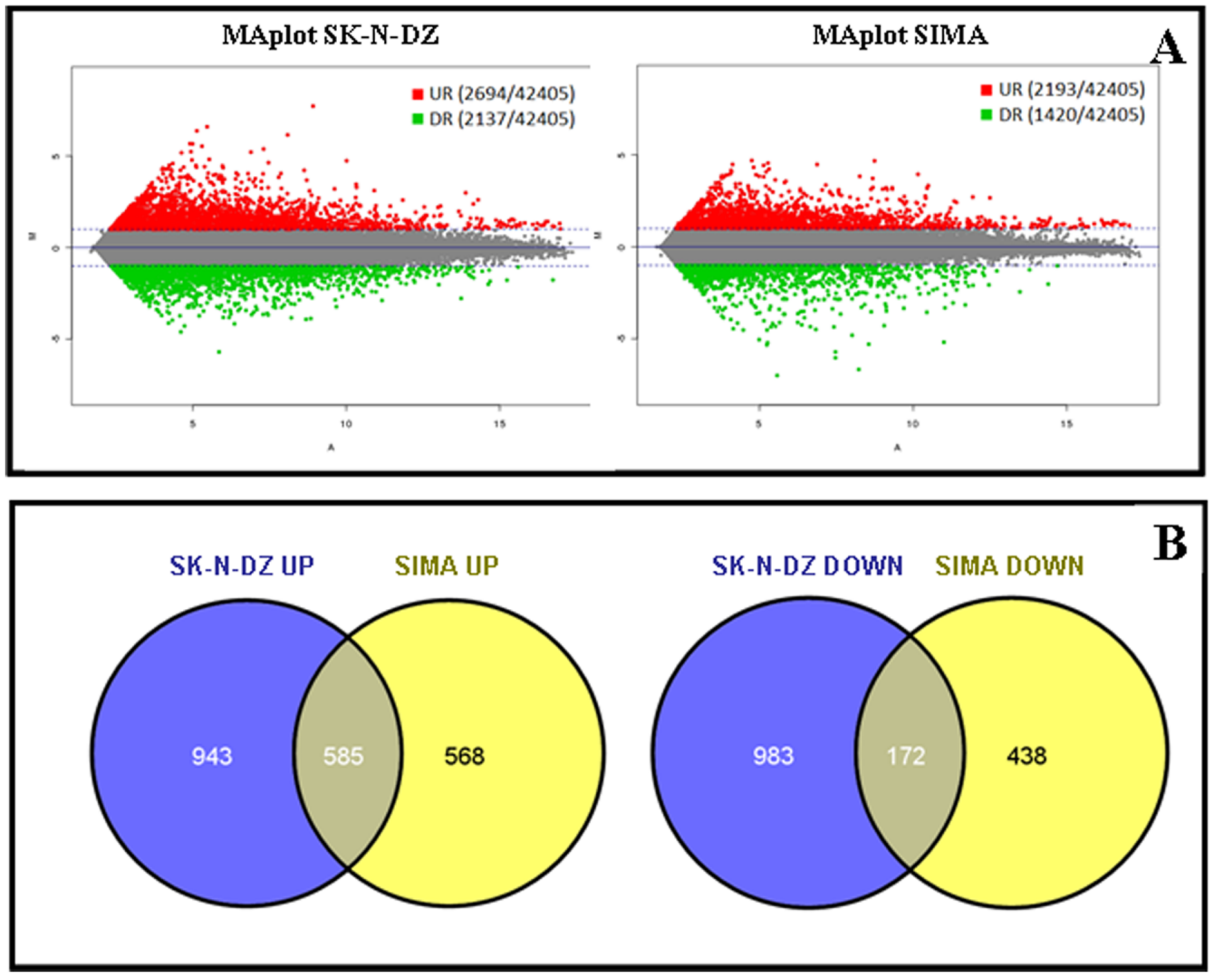
Upregulated (UR, red) and downregulated (DR, green) genes (>1 fold-change) in neurospheres compared to standard cells lines SK-N-DZ and SIMA (A), represented with MAplots, were the Y-axis represents the red/green intensity ratio “M” and the X-axis the average intensity “A”. Venn diagrams illustrating the number of differentially expressed genes in SK-N-DZ and SIMA neurospheres compared to standard cell lines (B). The overlapping area represents the set of genes altered in both cell lines.

In order to characterize the differentially expressed genes in CSC-like compared to the standard tumor cell line, mRNA expression was analyzed by microarray analysis. After the first intensity filter, 25,368 genes out of the whole dataset of 42,405 (∼60%), reached the minimum quality threshold and were selected for the differential expression analysis. When setting a threshold value of >1 fold-change, 4,831 genes (∼11.4%) were identified as differentially expressed in SK-N-DZ neurospheres and 6,613 genes (∼15.6%) in SIMA neurospheres. Figure 2A shows the number of genes differentially up and downregulated in each cell line. Among these gene sets, only 757 genes have significantly changed expression in both cell lines (Figure 2B). The sets of genes up and dowregulated for each cell line are summarized in Table S1 and the genes shared by both cell lines are represented in Tables S2, S3 and S4.

Differentially expressed genes with >1 fold-change in microarray analysis were categorized according to the biological processes they are involved in, and to the molecular functions they code for, by using the DAVID database. Among all categories, those that appeared significantly altered or enriched (p<0.05 and more than 2 genes differentially expressed in both cell lines by category) were selected for further studies (Table 4).

**Table 4:** DAVID generated categories.

	# Genes	p value	Genes ID
**Biological processes (GO)**			
GO:0042127∼regulation of cell proliferation	30	3.97×10^−4^ (**)	*FGFR2, NAMPT, ACVRL1, FGFR3, ERBB3, PPARG, MITF, TGFB3, TNFSF12, JAG1, FLT3LG, EDNRA, CD9, S1PR3, INS, TGM2, TESC, LYN, PTPRF, SF1, PTPRU, DHRS2, TNFSF13B, ID2, DBP, CHRM1, MYO16, PDGFRB, PTCH1, MCTS1*
GO:0007179∼transforming growth factor beta receptor signalling pathway	7	8.85×10^−4^ (**)	*AMHR2, SMAD9, ACVRL1, ID1, SMAD6, TGFB3, BAMBI*
GO:0000165∼MAPKKK cascade	11	2.51×10^−3^ (**)	*NRTN, DUSP2, FGFR3, ADORA2B, INS, LRRN3, TGFB3, ROR2, TNFRSF19, FGD4, DUSP6*
GO:0030509∼BMP signalling pathway	5	9.15×10^−3^ (**)	*SMAD9, ID1, SMAD7, SMAD6, GREM2*
GO:0042981∼regulation of apoptosis	24	2.91×10^−2^ (*)	*DEPDC6, KCNMA1, PTPRF, ERBB3, ARHGEF7, SMAD6, MITF, TGFB3, TNFSF12, STK3, CARD10, GCH1, ATP7A, DHRS2, KRT18, TNFSF13B, INS, ALDH1A3, BNIP3L, TGM2, TNFRSF19, BMF, FGD4, ANGPTL4*
GO:0051094∼positive regulation of developmental process	11	3.71×10^−2^ (*)	*TESC, SMAD9, ID2, PTPRF, LYN, INS, PPARG, TGFB3, TNFSF12, JAG1, ANGPTL4*
GO:0060393∼regulation of pathway-restricted SMAD protein phosphorylation	3	3.97×10^−2^ (*)	*SMAD7, SMAD6, TGFB3*
GO:0030155∼regulation of cell adhesion	7	4.45×10^−2^ (*)	*TESC, LAMA4, ARHGAP6, ACVRL1, ERBB3, SMAD7, TGM2*
GO:0043009∼chordate embryonic development	12	4.87×10^−2^ (*)	*C6ORF59, EDNRA, ACVRL1, HAND1, EPAS1, TGFB3, PDGFRB, ROR2, HES7, PTCH1, HOXD1, APBA1*
**Molecular Function (GO)**			
GO:0004629∼phospholipase C activity	5	3×10^−3^ (**)	*EDNRA, PLCB3, CHRM3, CHRM1, PLCB1*
GO:0004725∼protein tyrosine phosphatase activity	8	4×10^−3^ (**)	*DUSP5, DUSP2, PTPRF, PTPRH, PTPRT, PTPRU, RNGTT, DUSP6*
GO:0008081∼phosphoric diester hydrolase activity	7	5×10^−3^ (**)	*EDNRA, PLCB3, CHRM3, PDE7A, CHRM1, PDE4D, PLCB1*
GO:0034713∼type I transforming growth factor beta receptor binding	3	7×10^−3^ (**)	*SMAD7, SMAD6, TGFB3*
GO:0005072∼transforming growth factor beta receptor, cytoplasmic mediator activity	3	1.8×10^−2^ (*)	*SMAD9, SMAD7, SMAD6*
GO:0030695∼GTPase regulator activity	15	2.4×10^−2^ (*)	*ARHGEF7, EXPH5, DOCK9, RABGAP1L, SLC26A10, DOCK6, ARHGEF10, STARD13, MYRIP, ARHGAP6, SGSM2, SYTL4, CHN2, DOCK10, FGD4*
GO:0033549∼MAP kinase phosphatase activity	3	2.5×10^−2^ (*)	*DUSP5, DUSP2, DUSP6*
GO:0004714∼transmembrane receptor protein tyrosine kinase activity	5	4×10^−2^ (*)	*FGFR2, FGFR3, ERBB3, PDGFRB, ROR2*
GO:0005246∼calcium channel regulator activity	3	4.1×10^−2^ (*)	*NPY, HPCAL4, NPY2R*
GO:0005160∼transforming growth factor beta receptor binding	3	4.1×10^−2^ (*)	*SMAD7, SMAD6, TGFB3*

The differentially expressed genes in both cell lines were grouped in categories classified as significantly (*) and very significantly (**) enriched in neurosphere samples. Genes were categorized according to the biological processes they are involved in and to the molecular functions they code for.

**Figure 3:**
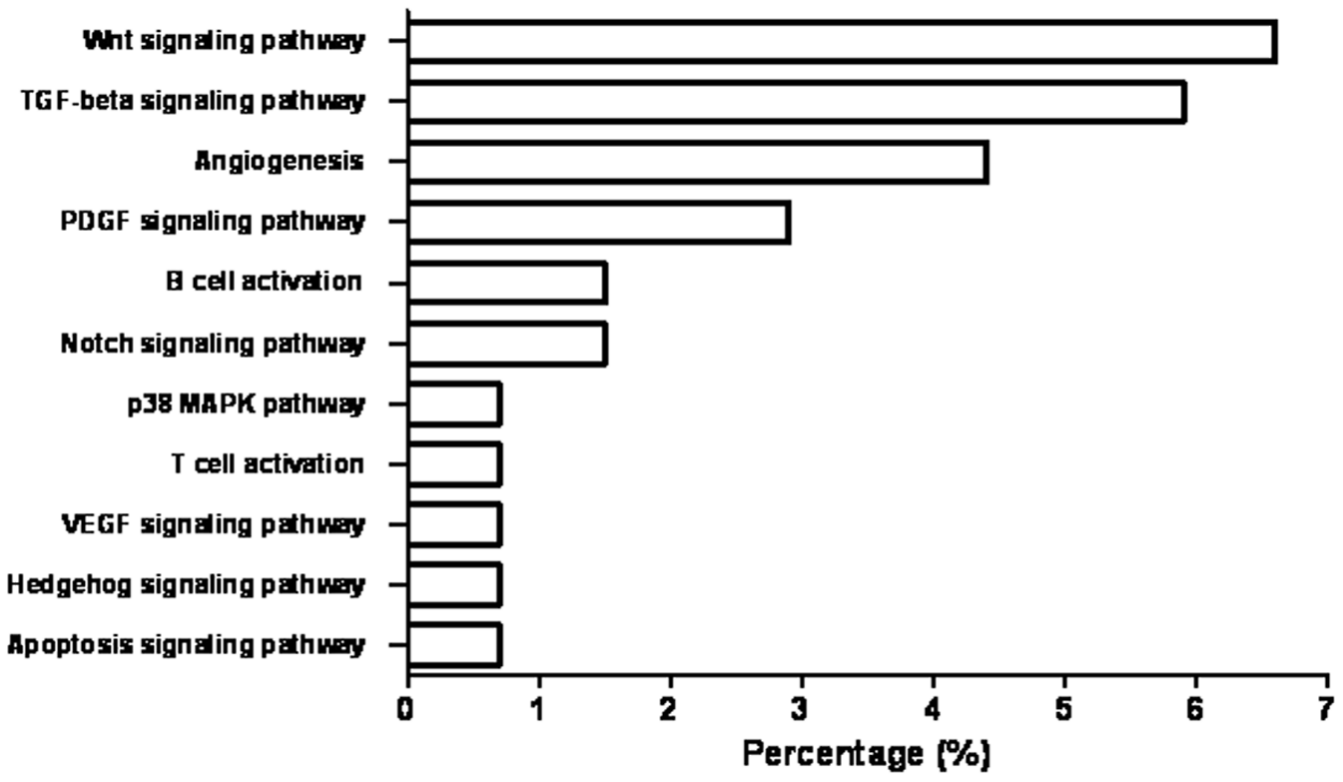
PANTHER classification by signalling pathway. The differentially expressed genes in both SK-N-DZ and SIMA CSC-like cells were classified by PANTHER and graphed. The percentage represents the number of genes altered against total number of genes involved in each pathway.

Further analysis of the significantly enriched categories and signalling pathways involved was performed with PANTHER database and information from the NCBI BioSystems database. As a result, we observed significant alteration in representative signalling pathways known to be also involved in stem cell self-renewal, as Wnt, Notch, Hh and TGF-β (Figure 3).

### Biological processes and signalling pathways expression in CSC-like subpopulation

The expression microarray experiment showed up a high percentage of genes altered in neurospheres (Figures 2 and 3). A complete analysis of the data highlighted some of the most relevant biological processes and molecular functions affected (Table 4) that might be responsible for the CSC-like phenotype of this subpopulation. First, we observed an apparent alteration of ion transporters. In our analysis, several genes of Ca^2+^ and K^+^ channels showed up as differentially expressed, as *KCNMA1* and *CACN1AG*. Secondly, we detected alterations in some of the most commonly studied signalling pathways in brain tumors and stem cell development: Wnt, Notch, Hh and TGF-β.

Analyzing Wnt signalling pathway, we observed downregulation of some of the upstream regulators of the pathway: *FZD8* (Frizzled receptor), *PRKCH* (protein kinase C) and *FRAT1*, inhibitor of GSK3-mediated phosphorylation of β-catenin, and therefore a positive regulator of the pathway that could act as a proto oncogene [30], [31]. The upregulation of *POU5F1* as a downstream target of the pathway was also noticed.

In Notch signalling pathway, overexpression of the activator ligand of the pathway, *JAG1* was identified. We also detected upregulation of *HES7*, one of the final targets of the pathway [32], [33]. This evidence showed an apparent activation of the pathway in CSC-like comparing with the standard cell line.

**Figure 4:**
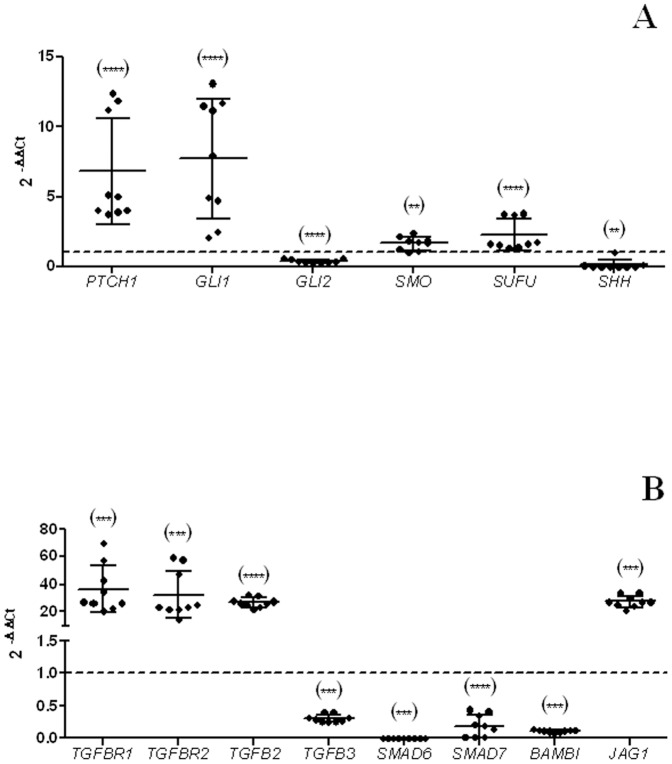
RT-qPCR for Sonic Hedgehog (A) and TGF-β pathways analysis (B) in SIMA CSC-like cells. The graphs represent the 2^−ΔΔCt^ values obtained by RT-qPCR for neurospheres. The dotted line indicates the 2^−ΔΔCt^ control (cell line) value equal to 1. Significance against control: p<0.05 (*); p<0.01 (**); p<0.001 (***) and p<0.0001 (****). Selected genes cover the most relevant components of each pathway. Results confirmed aberrant activation of Hh (A) and TGF-β (B) signalling pathways in CSC-like cells.

Expression microarray data showed upregulation of *PTHC1*, the main inhibitor of Hh signalling pathway, but also one of the gene targets of the pathway which transcription would be increased if the pathway is active, as we confirmed with the RT-qPCR data (Figure 4A).

Likewise, when analyzing TGF-β signalling pathway, the expression microarray data suggested an activation of the pathway (Figure 4B). Ligands like *TGFB3* and *BMP8B* were upregulated, as well as the signal transducer *SHC*. Quite the contrary, we found downregulation of inhibitory SMADs (like *SMAD6* and *SMAD7*) and the pseudoreceptor *BAMBI*, which could sequester ligands and inhibit signal transduction by different mechanisms [34].

### Parallel behaviour in SK-N-DZ and SIMA stem-like cells

**Figure 5:**
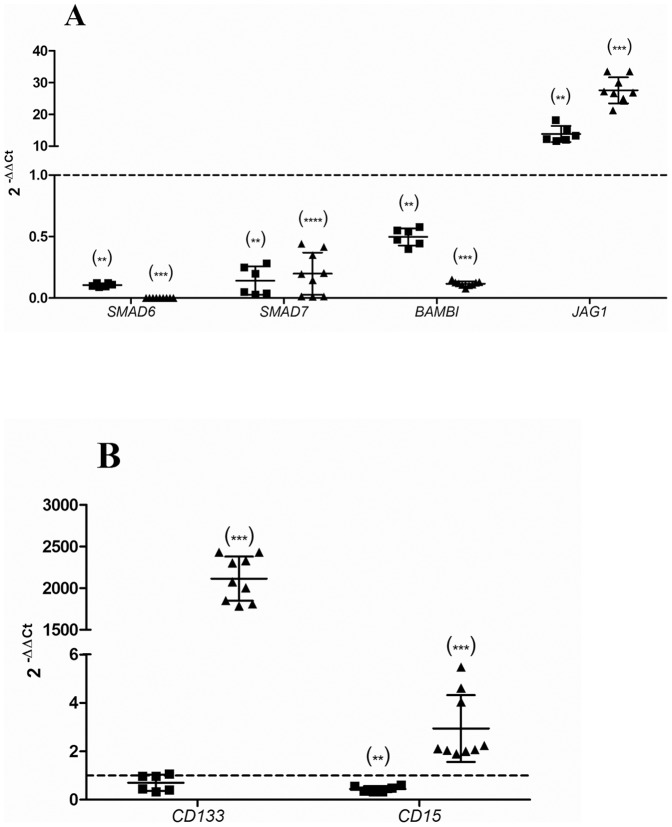
Gene expression by RT-qPCR. The graphs represent the 2^−ΔΔCt^ values obtained by RT-qPCR for neurospheres. The dotted line indicates the 2^−ΔΔCt^ control (cell line) value equal to 1. Significance against control: p<0.05 (*); p<0.01 (**); p<0.001 (***) and p<0.0001 (****). **Corroboration of expression array (A).** Both SK-N-DZ (▪) and SIMA (▴) CSC-like cells showed a similar expression profile. **Stem Cell Markers expression (B).** Results confirmed a high overexpression of CSC markers in SIMA cell line after the neurosphere formation assay.

RT-qPCR was performed in order to corroborate the expression microarray results. In both cell lines, we confirmed the decrease in the expression of *SMAD6*, *SMAD7* and *BAMBI*, as the increase observed in *JAG1*. The results suggested that the CSC-like subpopulation of both cell lines could have a similar expression profile after enrichment with the neurosphere assay (Figure 5A).

### Hh and TGF-β analysis in SIMA stem-like cells

Based on our previous experience in both Hh and TGF-β signalling pathways, further expression analysis by RT-qPCR was performed on some of the most relevant components of both signalling networks in SIMA cell line (Figure 4). Results confirmed Hh aberrant activation by overexpression of its key components as *PTCH1*, *GLI1* and *SMO*. Likewise, TGF-β seemed to be activated by overexpression of its receptors (*TGFBR1* and *TGFBR2*) and ligands (*TGFB2*), (despite *TGFB3* is downregulated even though it is also a ligand of the pathway), and downregulation of its repressors (*SMAD6*, *SMAD7* and *BAMBI*). We confirmed by RT-qPCR the increase of the levels of mRNA of *JAG1*, a Notch activating ligand that is related with TGF-β pathway [35]. Its upregulation supports Notch activation as aforementioned.

### Expression of stem-cell markers

To confirm the enrichment of the isolated population in CSC-like cells, analysis of CSC-specific cell surface markers was performed. RT-qPCR on *CD133* and *CD15* was carried out in both SK-N-DZ and SIMA neurospheres (Figure 5B). On the basis of these results, SIMA culture was highly enriched in CSC-like cells as shown by the significant increase in CSC surface markers expression. An interesting point is that SK-N-DZ subpopulation did not show any overexpression of the stem-cell markers analyzed.

## Discussion

The heterogeneous clinical behavior of neuroblastoma tumors has been widely studied [36]–[39]. It is already described that genomic rearrangements cannot explain per se this heterogeneity neither the CSC-like subpopulation [40]. In this work, we hypothesized that this heterogeneity might be due to genomic rearrangements together with changes in the expression profile of tumor cell subpopulations, particularly in CSC-like cells. In connection with this model, the genomic profile of CSC-like cells and the regulatory mechanisms or signalling pathways involved in CSC promotion could explain the heterogeneity and biology of this subpopulation and help to its identification in neuroblastoma.

Chromosomal aberrations are common in neuroblastoma, including numerical whole chromosomal gains, segmental chromosomal gains and losses and somatic mutations [2]. However, array-CGH did not show large modified areas in the genome of CSC-like subpopulations neither individually nor shared by both cell lines (Table 2). Despite this result, we identified other genetic elements involved in the biology of neuroblastoma and in the regulation of several tumors as CNV [41]–[43] and CpG islands [44]–[46] (Table 2). Both elements are being investigated as possible therapeutic targets in neuroblastoma [5] and were already identified in other tumors confirming their therapeutic potential [47]–[51]. These results suggest the fact that these two elements could be involved in tumorigenesis and CSC-like cells generation in neuroblastoma, and, as in other tumors, this might be a starting point to develop or improve current therapies. This is the case of decitabine (5-aza-2′-deoxycytidine). This agent interferes with DNA methylation but the preclinical assays show that it must be administrated at doses that are not tolerable in order to produce a biological effect [52]. Our findings propose a genetic basis for the effectivity of this kind of compounds in neuroblastoma highlighting the importance for further research in demethylating agents.

In addition, some microRNAs were identified in lost and gained areas (Table 3). In the last years, these elements have become promising therapeutic targets in neuroblastoma [53]. For example, in amplified loci of SIMA neurospheres, hsa-mir-4254 and hsa-mir-4262 were indentified (Table 3). These two microRNAs were already related with the stem cell phenotype [54], supporting the hypothesis that the CSC might be using the same regulatory machinery as normal stem cells [10], [11]. Other microRNA identified were hsa-mir-4420, hsa-mir-4429 and hsa-mir-4497, all described as possible new microRNAs involved in malignant human B cells regulation [55], and hsa-mir-3605, a novel microRNA linked to human cervical cancer [56].

In contrast, the expression array results indicated a modified expression pattern in CSC-like cells including changes in different processes and cell functions (Figures 2, 3 and Table 4). Different studies indicated some of the most important pathways involved in the development and maintenance of CSC subpopulation. Among them, the most remarkable are Wnt, Notch, Hh and TGF-β, which have been already described for different tumors and especially in brain tumors [14]. In neuroblastoma, Wnt and Notch genes have been identified as possible CSC-like markers. The difficulty in the identification of specific genes and accurate markers of CSC-like lies in the heterogeneity of this tumor and the poor knowledge related with this subpopulation, since no specific and definitive gene expression profile has been proposed for neuroblastoma CSC-like cells [57].

Interestingly, the expression pattern in CSC-like cells involves not only Wnt and Notch but also Hh and TGF-β signalling pathways. For example, deregulation of several genes of Wnt pathway was detected. The results indicated a decrease in the expression of upstream regulators as *FZD8*, *PRKCH* and *FRAT1* and an increase in the downstream target *POU5F1*, suggesting the inactivation of Wnt signalling pathway. *POU5F1* was described in several tumors (urothelial, prostatic, cervical, breast and lung) and is implicated in different functions as chemoresistance, prognosis marker, proliferation, apoptosis, migration and invasion [58]–[61]. In addition to all these functions, this gene plays an important role in stemness and self-renewal of stem cells in normal and tumor tissues [60], [62], [63] and was found to be overexpressed and correlated with the progression of neuroblastoma [64]. This gene could interact with members from other pathways as TGF-β, Notch and Wnt [65], [66] working together in the regulation of stem cell pluripotency. Our results show alterations not only in *POU5F1* but also in members of TGF-β, Notch and Wnt signalling pathways indicating the potential of this gene to be used as a new therapeutical target. Our findings are supported by other works that described its potential in other tumors [62], [64], [67] and as a possible new CSC marker in neuroblastoma [67]–[69].

In this context, an apparent activation of Notch pathway was detected in neurospheres. It has been demonstrated that inhibition of Notch in cancer cells has the potential to slow down cell proliferation and induce apoptosis, despite the extensive crosstalk of this pathway with other major cancer pathways as Ras, Akt, NF-κB, Wnt, Hh and TGF-β [70]–[72]. This might happen because in neuroblastoma as in other tumors, the CSC subpopulation seems to be especially sensitive to inhibition of stem cell pathways as Notch [73].

Additionally, Hh was activated in CSC-like cells by upregulation of transcription targets and activators (*PTCH1*, *SMO* and *GLI1*) regardless downregulation of *GLI2*. It is known that *GLI2* is a bi-functional transcription factor, with activator and repressor regions. Therefore the pathway could still be activated if *GLI2* is silenced depending on the context [74], [75]. In neuroblastoma CSC-like the Gli factors act through cooperative functional interactions in target gene regulation [76] although in our case it seems that *GLI2* does not play an important role. Alike, the TGF-β signalling pathway is activated in neurospheres cultures (Figure 4B) despite the downregulation of *TGFB3*, a ligand of the pathway. A possible explanation could be that the downregulation of *BAMBI* (Figure 4B) would let a greater proportion of ligands free, increasing the concentration of extracellular ligands and consequently facilitating the activation of the pathway or acting as a negative regulator of the TGF-β pathway [34]. Interestingly, *JAG1*, one of the targets of TGF-β [77] and also the main activator of Notch signalling pathway is upregulated, supporting Notch signalling pathway activation. Kurpinski *et al* described *JAG1* as a crosstalk point between these two pathways in the regulation of muscular stem cells [78] which probably suggests an important role of this gene in the generation of CSC-like cells in neuroblastoma. These findings also give us the possibility of studying targets as *JAG1* that participate in several genetic pathways involved in CSC-like cells phenotype.

It is clear that the expression profile that we observed in the CSC-like cells is similar to other profiles described for this subpopulation in different tumors including neuroblastoma. The identification of CSC is based on the expression of different markers, but in the case of neuroblastoma it remains unclear which markers are accurate to identify this subpopulation, due to its heterogeneity. Our findings show differences between the two cell lines used (Figure 5B) in two CSC markers widely described: *CD133* and *CD15*. This result could highlight the limitations of neurospheres culture [79] since it was proved that differences in the culture conditions could affect the enrichment and isolation of CSC-like cells [80]. However, since the genes related with CSC followed a similar pattern in both cell lines, the more plausible explanation is that the phenotype of CSC-like cells is not yet fully characterized in neuroblastoma [57]. Coulon *et al* [57] questioned the expression of CD133 to identify CSC in neuroblastoma, highlighting the need of characterization of other CSC markers or expression profiles that allow the recognition of this subpopulation. The choice of CSC markers is still controversial and the search of specific genes that help to identify it is the real challenge for the characterization and research of new therapeutical targets in neuroblastoma [81].

Regardless the growing evidence in new approaches and in the identification of new therapeutic targets, the treatment for neuroblastoma is still evasive. Nowadays, it includes surgery, radiation and/or chemotherapy depending on the patient's stage and risk stratification of the disease [5]. Only some new compounds are included in the schema as 13-cis-retinoic acid together with anti-GD2 antibodies and interleukin-2, a combination that has increased the progression-free survival [82]. The literature includes an increasing number of new compounds with great potential to be introduced in the treatment. This is the case of drugs that targeted *MYCN* and *ALK* inhibitors that currently are in preclinical evaluation [83]. The evidence of possible targets involved in the development and regulation of other tumors could be an approach to develop new strategies in neuroblastoma. A good example are the different pathways involved in tumorigenesis and CSC regulation, as Wnt [62], [64], [67]–[69], Notch [84], Hh [85]–[87] and TGF-β [18], [88], [89]. In this connection, an interesting case are the ion transporters, described as essential for cell proliferation and which appear to have a role in cancer development [90]–[92]. Evidence is particularly extensive for Ca^2+^ and K^+^ channels [93] and Romania *et al* already described their importance in neuroblastoma [94]. In our analysis, several genes coding for Ca^2+^ and K^+^ channels were altered in CSC-like subpopulation like *KCNMA1*, previously described in prostate cancer [95] and in breast cancer [96], [97], and *CACN1AG* also altered in gastric cancers, colorectal cancers and acute myeloid leukemia (AML) [98]. Several reports propose that ion transporters have clinical potential not only as therapeutical targets but also as prognosis markers or to improve actual treatments [99]–[103], as blocking channel activity seems to impair the growth of some tumors, including neuroblastoma [99], [104]. The presence of altered genes of Ca^2+^ and K^+^ channels in neuroblastoma CSC-like subpopulations confirms and opens the possibility to develop new compounds that could act at different levels and subpopulations within neuroblastoma. This is only one example of the importance of redirecting treatments towards more effective CSC molecular therapies to achieve total elimination of the tumor cell population and avoid relapses [40], [105].

In conclusion, we found that the expression profile of neuroblastoma CSC-like cells differs significantly from the expression profile in standard neuroblastoma cell lines, which is seen as altered signalling pathways involved in stem cell proliferation pathways as Wnt, Notch, Hh and TGF-β, suggesting a cross talk among them and with other pathways. The CSC markers analysis reveals no correlation between the cell lines, displaying the heterogeneity of neuroblastoma tumors and CSC-like population but opening the possibility to identify other potential markers. Taking together, these results confirm the importance of different pathways in CSC regulation and the identification of possible candidate targets for molecular CSC therapies in neuroblastoma. The results presented highlight new information for the CSC-like phenotype in neuroblastoma and indicate the importance of redirecting current cancer treatments towards CSC molecular therapies to achieve total elimination of the tumor cell population and improve treatment effectiveness.

## Supporting Information

Table S1 **Supplementary data.** Up and downregulated genes list from the expression array in each cell line.
(XLS)

Table S2 **Supplementary data.** Upregulated genes list shared in both cell lines.
(XLS)

Table S3 **Supplementary data.** Downregulated genes list shared in both cell lines.
(XLS)

Table S4 **Supplementary data.** Genes list not found in PANTHER classification.
(XLS)
